# Classification of Smoking Cessation Apps: Quality Review and Content Analysis

**DOI:** 10.2196/17268

**Published:** 2022-02-17

**Authors:** Suin Seo, Sung-Il Cho, Wonjeong Yoon, Cheol Min Lee

**Affiliations:** 1 Department of Epidemiology, Graduate School of Public Health Seoul National University Seoul Republic of Korea; 2 Department of Family Medicine Healthcare System Gangnam Center Seoul National University Hospital Seoul Republic of Korea

**Keywords:** smoking cessation, app, type, content and functions, MARS, quality, score, mobile phone

## Abstract

**Background:**

Many people use apps for smoking cessation, and the effectiveness of these apps has been proven in several studies. However, no study has classified these apps and only few studies have analyzed the characteristics of these apps that influence their quality.

**Objective:**

The purpose of this study was to analyze the content and the quality of smoking cessation apps by type and identify the characteristics that affect their overall quality.

**Methods:**

Two app marketplaces (App Store and Google Play) were searched in January 2018, and the search was completed by May 2020. The search terms used were “stop smoking,” “quit smoking,” and “smoking cessation.” The apps were categorized into 3 types (combined, multifunctional, and informational). The tailored guideline of Clinical Practice Guideline for Treating Tobacco Use and Dependence was utilized for evaluating app content (or functions), and the Mobile App Rating Scale (MARS) was used to evaluate the quality. Chi-square test was performed for the general characteristics, and one-way analysis of variance was performed for MARS analysis. To identify the general features of the apps that could be associated with the MARS and content scores, multiple regression analysis was done. All analyses were performed using SAS software (ver. 9.3).

**Results:**

Among 1543 apps, 104 apps met the selection criteria of this study. These 104 apps were categorized as combined type (n=44), functional type (n=31), or informational type (n=29). A large amount of content specified in the guideline was included in the apps, most notably in the combined type, followed by the multifunctional and informational type; the MARS scores followed the same order (3.64, 3.26, and 3.0, respectively). Regression analysis showed that the sector in which the developer was situated and the feedback channel with the developer had a significant impact on both the content and MARS scores. In addition, problematic apps such as those made by unknown developers or copied and single-function apps were shown to have a large market share.

**Conclusions:**

This study is the first to evaluate the content and quality of smoking cessation apps by classification. The combined type had higher-quality content and functionality than other app types. The app developer type and feedback channel with the app developer had a significant impact on the overall quality of the apps. In addition, problematic apps and single-function apps were shown to have a large market share. Our results will contribute to the use and development of better smoking cessation apps after considering the problems identified in this study.

## Introduction

Smoking is a repetitive addictive behavior [1,2], and environmental conditions promoting its cessation are important. A smoke-free environment achieved through antismoking campaigns and use of assistive devices such as computers and smartphones throughout the day could be helpful [3-5]. In particular, smartphones allow continuous monitoring of smoking and facilitate cessation because users have access to them at all times. Approximately 8.2 billion mobile cellular telephones, including smartphone subscriptions, were reported worldwide in 2020, which exceeds the world’s population [6]. Apps are the most common features of smartphones. In the first quarter of 2021, Google Play had 3.48 million apps available for download and the iPhone App Store had 2.22 million apps [7]. In the first quarter of 2021, the total downloads from App Store and Google Play app amounted to an estimated 36.6 billion [8]. This illustrates the degree to which smartphone apps are used. Thus, a firmly established smoking cessation app could have a major impact.

Smartphone apps have been increasingly used for promoting behavioral changes and have proved to be effective with regard to physical activity and noncommunicable diseases. They greatly influence people’s daily lives, particularly with respect to goal-based behavioral modifications [9-11]. As the number of people using a smartphone app to quit smoking has increased, there has been a concomitant increase in the number of smoking cessation apps available [12]. In a comprehensive review of studies evaluating the effects of smoking cessation apps, it was found that an evidence to determine the effect of the apps was not enough [13]. However, a few smoking cessation apps have been proven to be effective. Some studies have found the effects of individual apps with distinct characteristics. Smokers who use decision-aid apps are more likely to be continuously abstinent compared to those using information-only apps at 1 month (relative risk [RR] 1.68, 95% CI 1.25-2.28), 3 months (RR 2.08, 95% CI 1.38-3.18), and 6 months (RR 2.02, 95% CI 1.08-3.81) [14]. An evidence-based app with customized functions and information is more effective than a web-based self-help booklet for smoking cessation [15]. Thus, apps providing information and various functions are more effective than apps that provide information only.

Although many studies have evaluated smoking cessation apps and compared the effects between individual apps with distinct characteristics, no study has classified all the apps by their characteristics. The objective of this study was to evaluate the content and quality of smoking cessation apps by type and determine which type of apps have better content and quality. Furthermore, we aimed to identify the app characteristics associated with the content (or functions) and quality thereof. To this end, first, we identify the types of smoking cessation apps. Second, we determine which type of smoking cessation apps have more accurate content and high quality for continuous use.

## Methods

### App Search Strategy

Two app markets (App Store and Google Play), which account for over 80% of all the available mobile apps, were searched for this study [16]. English-language apps available to Koreans were searched by using the terms “stop smoking,” “quit smoking,” and “smoking cessation,” as done in previous studies [12,17-19]. The app search was completed by May 2020.

### App Selection

We reviewed all the smoking cessation apps that we obtained with our search terms. We excluded apps that were not concerned with smoking cessation, not in English, not designed for smartphones (eg, Tablet and iPad apps), or not showing proper functionality, as well as those targeting specific groups, designed for commercial purposes, related to the overall health behavior and not just smoking cessation, or including only photos, videos, or games. As the purpose of this study was to compare apps by function, single-function apps that have only 1 function (eg, counter or hypnosis) were excluded. Apps with only 2 assistive functions or 1 function plus information were also excluded from the analysis because the functions of these apps were very limited similar to single-function apps. The main function of these apps is “counter (tracker)” and the other function is very minor (eg, free notes, little information, unidentified chatting). Since these criteria are not applicable in many items of evaluation tools, evaluation has no meaning. In the analysis of content and function, questions for an “advise” category, an “assess” category, and an “assist category-support provided” do not apply to single-function apps (eg, personalized advice, user could indicate lack of readiness to quit, users could interact with other users for mutual support [app community]). In the analysis of quality (using Mobile App Rating Scale [MARS]), some questions of the information quality category do not apply to single-function apps (eg, Credibility: Does the app come from a legitimate source? specified in App Store description or within the app itself). Apps that met our criteria were included in the final analysis (Multimedia Appendix 1).

### App Categorization

The apps were categorized into 3 types at the eligibility assessment stage by reference to prior studies [14,15]. Those 2 studies compared information-only apps (self-help booklets) with apps, including both information and specific functions to motivate the user to stop smoking. The effectiveness of the information-only apps for quitting smoking was lower than that of the apps consisting of both information and specific functions. It is necessary to classify the overall smoking cessation apps as a feature confirmed in the individual app effect evaluation. Therefore, in this study, we initially classified apps into 2 types: information-only apps and apps with functions. The availability of information has a significant impact on health behavior changes [11]. The apps with functions were further subdivided into apps that provided information and apps that did not, thereby resulting in 3 categories of apps in our study. The 3 categories were formally designated as informational, multifunctional, and combined type. Informational apps provide information only similar to an electronic book and have no function (eg, counters, alarms, games, community features). Multifunctional apps have 3 or more assistive functions but provide little information. Combined apps provide both information and at least 2 assistive functions.

### Critical Appraisal of the Apps (Quality Assessment)

Two evaluation tools were used to assess the apps. A tailored guideline [20] for use of the 5As (Ask, Advise, Assess, Assist, and Arrange follow-ups), as recommended in the Clinical Practice Guideline for Treating Tobacco Use and Dependence [21], was modified to evaluate the content and functions of the apps [22]. The MARS was used to evaluate the functional quality of the apps [23]. The apps were rated by 2 independent researchers using a standardized rating form. After using each app for a minimum of 15 minutes, raters evaluated them twice, that is, once each using a content and function analysis form and a MARS form. Apps on both markets were analyzed using both iPhones and Android phones for including all functions. Each assessment took approximately 40-50 minutes. The consistency of the assessments was measured according to the interrater reliability. Discrepancies between content analyses were resolved by consensus between the 2 raters. This consensus process was not necessary for the MARS analysis because the MARS score was the average of the 2 assessments.

### Analysis of Content and Function

The revised guideline for evaluating apps [20] from the Clinical Practice Guide for Treating Tobacco Use and Dependence [21] was used for content and function analysis. This guideline breaks down the interventions into 5 major parts: ask, advise, assess, assist, and arrange follow-ups (the 5As). In this study, in the “ask” category, the smoking status of the users was confirmed. In the “advise” category, the raters identified whether or not the app included advice on quitting smoking. In the “assess” category, the raters identified whether the app possessed a function to evaluate the user’s readiness for quitting smoking. In the “assist” category, the various functions of the apps designed to help users quit smoking were evaluated. Finally, in the “arrange follow-ups category,” raters identified whether the app could track the user’s smoking cessation status. By referring to the World Health Organization’s “A guide for tobacco users to quit” [22], we added new content to the “assist” category, namely, social benefits, health risk of smoking, and confidence (motivation). The modified guideline for content and function analysis consisted of 39 questions. Each question used a yes/no scale; therefore, the total possible score was 39 points. The interrater reliability between 2 raters was assessed and the kappa value was 0.75.

### Analysis of Quality (Using MARS)

The MARS is a mobile health (mHealth) app quality assessment tool that provides a multidimensional measure of app quality according to the following indicators: engagement, functionality, aesthetics, and information quality. It is a 23-item, expert-based rating scale that can be used to systematically evaluate the quality of mHealth apps on a 5-point scale (1=inadequate, 2=poor, 3=acceptable, 4=good, and 5=excellent) [23]. The engagement category is concerned with factors such as fun, interest, customizability, interactivity (eg, sending alerts, messages, reminders and feedback, sharing), and suitability of material. The functionality category is concerned with technical functions such as functioning, ease of learning, navigation, flow, logic, and gestural design. The aesthetics category subsumes graphic design, visual appeal, color scheme, and stylistic consistency. Information quality is concerned with whether the information (eg, text, feedback, measurements, references) is from a credible source. The interrater reliability between the 2 raters was assessed by the intraclass correlation and had a value of 0.6 (95% CI 0.4-0.74).

### Statistical Analyses

General characteristics were analyzed by the chi-square test. Each dimension on MARS was analyzed by one-way analysis of variance The analyses of content score (ie, the score of content and function analysis) and MARS scores (ie, the score of MARS analysis) were conducted by app type. To identify characteristics of apps associated with the MARS and content scores, multiple regression analysis was performed. The sector in which the developer was situated, whether they had an affiliation with health care professionals, the app platform, payment type, and feedback type represented the characteristics of interest. All analyses were performed using SAS software (ver 9.3; SAS Institute).

## Results

### General Characteristics of the Apps by Type

**Figure 1:**
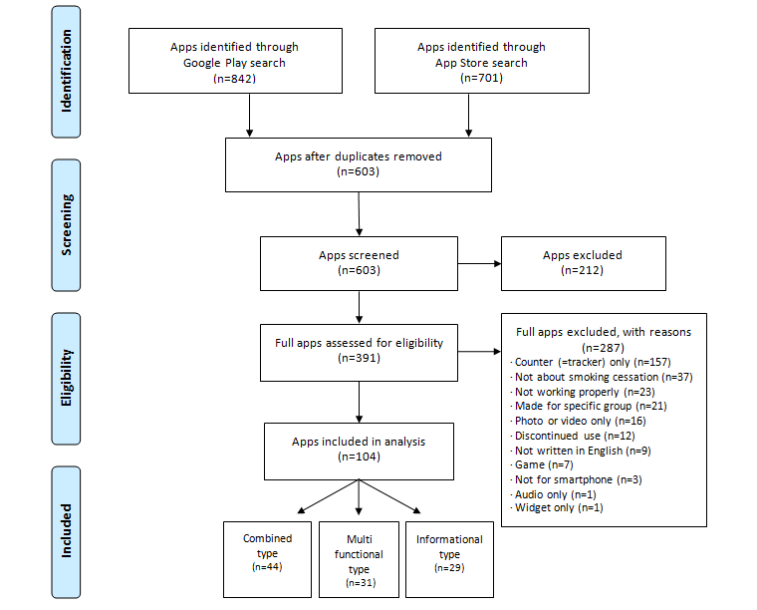
Study flow diagram.

A total of 1543 apps (App Store, n=701; Google Play, n=842) were identified via the search terms, of which 940 duplicated apps were excluded. Thus, 603 apps (App Store, n=305; Google Play, n=260; both markets, n=38) were preliminarily screened, and 212 irrelevant apps were excluded. The remaining 391 relevant apps (App Store, n=174; Google Play, n=181; both markets, n=36) were screened according to selection criteria. Finally, 104 apps (App Store, n=30; Google Play, n=39; both markets, n=35) were included in the analysis (Figure 1). Table 1 shows the general characteristics of the apps by type. Of the 104 apps assessed, there were 44 combined apps, 31 multifunctional apps, and 29 informational apps.

**Table 1:** General characteristics of the apps by type (N=104).

Category, items			Combined type (n=44)		Multifunctional type (n=31)		Informational type (n=29)		*χ* ^2^ *(df)*		*P* value	
**Platform, n (%)**										18.1 (4)		.001
	Android only	10 (23)		14 (45)		15 (52)						
	iPhone only	12 (27)		6 (19)		12 (41)						
	Both	22 (50)		11 (36)		2 (7)						
**Last update^a^, n (%)**										14.3 (8)		.07
	<1 month	7 (16)		5 (16)		0 (0)						
	1-6 months	8 (18)		7 (23)		4 (14)						
	6-12 months	13 (30)		9 (29)		7 (24)						
	>12 months	14 (32)		8 (26)		10 (34)						
	Unknown	2 (5)		2 (7)		8 (28)						
Advertisements in the app, n (%)			9 (20)		14 (45)		17 (59)		14.3 (4)		.007	
**Payment type, n (%)**										25.6 (4)		<.001
	Free	29 (66)		16 (52)		19 (66)						
	In-app purchase	15 (34)		10 (32)		0 (0)						
	Prepaid	0 (0)		5 (16)		10 (34)						
Price (prepaid app)^b^, mean (SD)			N/A^c^		2330 (2730)		3910 (8360)		N/A		N/A	
**Developer sector, n (%)**										57.6 (10)		<.001
	Government or university	13 (30)		0 (0)		1 (3)						
	Government with commercial	4 (9)		0 (0)		0 (0)						
	Commercial	21 (48)		20 (65)		4 (14)						
	Nongovernment organization	0 (0)		2 (6)		0 (0)						
	Unknown	6 (14)		9 (29)		24 (83)						
Affiliation of developer with health care professionality, n (%)			25 (57)		2 (6)		1 (3)		34.7 (2)		<.001	
**Feedback channel with developer, n (%)**										20.6 (4)		<.001
	Within the app	19 (43)		13 (42)		2 (7)						
	Contact information of developer provided	11 (25)		3 (10)		3 (10)						
	Market level only	14 (32)		15 (48)		24 (83)						

^a^Updated on May 2020.

^b^US $1=1100 won; the cost of in-app purchases was calculated as the average cost if prices differed among items.

^c^N/A: not applicable.

The platform, presence of advertisements in the app, payment type, developer sector and health professional affiliation status, and feedback channel with developer showed significant differences by app type. Regarding differences in platforms, of the 44 combined apps, 22 (50%) were available in both Google Play and the App Store. Of the remainder, more were available in the App Store (12/44, 27%) than in Google Play (10/44, 23%). Of the 31 multifunctional apps, 11 (36%) were available in both markets. The remainders were more frequently available in Google Play (14/31, 45%) than in App Store (6/31, 19%) in contrast to the combined type. Informational apps were least frequently available in both markets (2/29, 7%); 15 (52%) of the 29 informational apps were available in Google Play only, and 12 (41%) were available in the App Store only. Advertisements were found in 17 (59%) of the 29 informational type, 14 (45%) of the 31 multifunctional type, and 9 (20%) of the 44 combined type. With respect to payment for the apps, all of the 44 combined apps were initially free, of which 15 (34%) required payment within the app to utilize all functions (in-app purchase). Regarding the 31 multifunctional apps, 5 (16%) were purchased and 26 (84%) were free. Of the 31 free multifunctional apps, 10 (32%) had in-app purchase functionality. Regarding the 29 informational apps, 10 (34%) were prepaid, and in contrast to the other 2 categories of apps, none of the free informational apps (19/29, 66%) had in-app purchase functionality.

Regarding developer sector, for the combined type, the largest proportion of the apps was developed within the private sector (21/44, 48%) followed by government or university independently (13/44, 30%) and then by collaboration between the government and a professional app development company (4/44, 9%). For 6 (14%) of the 44 combined type apps, no developer was identified. Regarding the 31 multifunctional apps, the majority were commercially developed (20/31, 65%), and only 2 (6%) apps were developed by nongovernment organizations. For nearly a third (9/31, 29%) of the multifunctional apps, the sector in which they were developed was not known. For informational apps, only a small proportion of the developers identified were commercial developers (4/29, 14%) and only 1 app (1/29, 3%) was developed by government. For the majority of the informational apps (24/29, 83%), the affiliation of the developer was unknown. Apps developed by health professionals were most prevalent in the combined type (25/44, 57% vs 2/31, 6% and 1/29, 3% for the multifunctional and informational type, respectively).

When we assessed the apps in terms of the feedback channel with the developer, a significant difference between app types was seen again. Among the combined type, apps that had an option to communicate with a developer within the apps were the most common (19/44, 43%); 14 (32%) did not have the option and communication was only possible at the market level (ie, Google Play or App Store) and 11 (25%) included the developer’s contact information within the apps or as a link. Among the multifunctional type, the largest proportion (15/31, 48%) of the apps enabled communication with a developer only at the market level, while 13 (42%) provided the option to communicate using the apps and 3 (10%) included the developer’s contact details within the apps or as a link. Among the informational type, the vast majority of apps enabled communication with a developer only at the market level (24/29, 83%); only 3 (10%) included the developer’s contact details within the apps or as a link, and 2 (7%) apps enabled communication with a developer directly through the apps.

### Content and Function of the Apps by Type

Table 2 shows how well the content and functions of the apps matched the guideline. For all 5As components, the content of combined apps was the most consistent with the guideline. Regarding the “ask,” “assist,” and “arrange follow-up” components, multifunctional apps were more consistent with the guideline than informational apps. Regarding the “advice” and “assess” components, informational apps were more consistent with the guideline than multifunctional apps. All apps addressed the “assist” component. “Arrange follow-ups” was offered in some respect by the 44 combined apps (44/44, 100%), 30 multifunctional apps (30/31, 97%), and 10 informational apps (10/29, 34%); a substantial proportion of the apps asked smoking status (ask; 44/44, 100%; 30/31, 97%; and 0/29, 0%; respectively) and offered advices to quit smoking (advice; 38/44, 86%; 11/31, 35%; and 29/29, 100%; respectively), but very few fulfilled the “assess” component (23/44, 52%; 1/31, 3%; and 18/29, 62%; respectively), that is, readiness to change and interest in quitting.

**Table 2:** Content and functions of the apps by type (N=104).

Function				Combined type (n=44), n (%)		Multifunctional type (n=31), n (%)		Informational type (n=29), n (%)
**Ask (app assessed smoking status)**								
	Overall			44 (100)		30 (97)		0 (0)
	**Current smokers**							
		Number of cigarettes smoked per day	41 (93)		29 (94)		0 (0)	
		Time until first cigarette of the day	9 (20)		3 (10)		0 (0)	
		Smoke when sick	6 (14)		2 (6)		0 (0)	
		Reasons to smoke/quit smoking	22 (50)		4 (13)		0 (0)	
	**Smoking triggers**							
		Time of day-related smoking triggers	21 (48)		4 (13)		0 (0)	
		Other smoking triggers	13 (30)		4 (13)		0 (0)	
**Advise (app advised the user to quit smoking)**								
	**Overall**							
		General advice	38 (86)		11 (35)		29 (100)	
		Personalized advice (using user-provided info)	8 (18)		2 (6)		0 (0)	
**Assess (app assessed the user’s readiness to quit)**								
	**Overall**			23 (52)		1 (3)		18 (62)
		User could indicate lack of readiness to quit	14 (32)		1 (3)		0 (0)	
		Barriers to quitting were addressed^a^	23 (52)		1 (3)		18 (62)	
**Assist (app assisted the user with the quit attempt)**								
	Overall			44 (100)		31 (100)		29 (100)
	**Setting a quit date**							
		Users were asked to pick a quit date	41 (93)		24 (77)		0 (0)	
		Users received support for their quit attempt	44 (100)		30 (97)		7 (24)	
		Users received feedback on their quit attempt^b^	4 (9)		1 (3)		0 (0)	
	**Reward**							
		Reminders about money saved since quitting	40 (91)		23 (74)		0 (0)	
		Reminders about health benefits accrued	19 (43)		22 (71)		10 (34)	
		Information about social benefits^b^	13 (30)		4 (13)		5 (17)	
	**Risk**							
		Information about health risks of smoking^b^	27 (61)		7 (23)		9 (31)	
	**Support provided**							
		Distraction from urges, reminders about number of cigarettes not smoked since quitting	43 (98)		29 (94)		10 (34)	
		Users could interact with other users for mutual support (app community)^a^	12 (27)		12 (39)		0 (0)	
		Web community^b^	2 (5)		2 (6)		0 (0)	
		Referral to Quitline or other support groups	18 (41)		3 (10)		3 (10)	
		Recorded personalized message to be played back later	24 (55)		18 (58)		0 (0)	
		Reminders of their motivations during difficult times	19 (43)		13 (42)		0 (0)	
		Motivation alarm^b^	17 (39)		4 (13)		0 (0)	
		Personalized motivation alarm^b^	4 (9)		3 (10)		0 (0)	
		Encouragement (to improve self-confidence, helpful quotes)^b^	20 (45)		8 (26)		10 (34)	
	**Information provided**							
		Information on self-confidence^b^	4 (9)		0 (0)		13 (45)	
		Information on counseling, treatment, meds, Quitline, etc	42 (95)		1 (3)		29 (100)	
		Links to resources	19 (43)		1 (3)		2 (7)	
**Arrange follow-ups (app followed up with the user regarding the quit attempt)**								
	**Overall**			44 (100)		30 (97)		10 (34)
		Checked-in prior to quit attempt	30 (68)		17 (55)		0 (0)	
		Checked-in after quit attempt	44 (100)		20 (65)		2 (7)	
		If relapsed, encouraged user to set a new quit date	34 (77)		27 (87)		1 (3)	
		If relapsed, possible to add smoking number^a^	19 (43)		7 (23)		0 (0)	
		If relapsed, offered encouragement that quitting takes practice	7 (16)		1 (3)		0 (0)	
		Information provided about relapse^b^	9 (20)		1 (3)		8 (28)	

^a^Modification of the original tool.

^b^New additions to the original tool.

The “ask” component could not be addressed by the informational type, which provides information only and has no specific function. In terms of the questions for the current smokers, most apps in the other two types asked about the number of cigarettes smoked per day (41/44, 93% of combined apps; 29/31, 94% of multifunctional apps), but very few apps in those types asked about smoking when sick (6/44, 14% and 2/31, 6%, respectively). Few multifunctional apps asked about the smoking triggers (4/31, 13%). The advice provided by the apps was largely generic and very rarely personalized (8/44, 18% of combined apps; 2/31, 6% of multifunctional apps; and none of the informational apps). Barriers to readiness to quit was assessed or explained in nearly a half of the combined apps (23/44, 52%), 18 (62%) of the 29 informational apps, and 1 (3%) of the 31 multifunctional apps. However, there were almost no apps in which users could indicate their barriers on their own, except for the combined apps (combined apps 14/44, 32%; multifunctional apps 1/31, 3%; and informational apps 0).

Among the combined apps, the “assist” content was typically in the form of basic support regarding quit attempts (44/44, 100%; at least one function facilitating smoking cessation), reminders about the number of cigarettes not smoked or days of not smoking (43/44, 98%), asking the user to pick a quit date (41/44, 93%), and basic information on smoking cessation (42/44, 95%; eg, electronic books providing facts about smoking). Assist-related functions that could exploit smartphone technology to provide personalized content such as a personalized motivation alarm (4/44, 9%), tailored feedback on quit attempts (4/44, 9%), or information promoting self-confidence (4/44, 9%) were rarely utilized. Among the multifunctional apps, the “assist” content commonly consisted of basic support regarding quit attempts (30/31, 97%) and reminders about the number of cigarettes not smoked or days of not smoking (29/31, 94%). Similar to the combined apps, personalized feedback on quit attempts (1/31, 3%) and a personalized motivation alarm (3/31, 10%) were rarely utilized. Additionally, few apps provided information on basic smoking cessation information (1/31, 3%) and links to resources (1/31, 3%).

Among informational apps, basic information was provided by 29 (100%) of the apps, and 10 (34%) included general information about self-confidence, the health benefits of cessation, and the number of cigarettes smoked or days of not smoking. Less than 5 (17%) of the 29 informational apps provided information about social benefits, support regarding the user’s quit attempt or Quitline or other support groups, or links to resources.

The “arranging follow-up” component, which only apps can offer, typically consist of a “check-in” prior to a quit attempt (30/44, 68% of combined apps; 17/31, 55% of multifunctional apps), check-in after a quit attempt (44/44, 100% and 20/31, 65%, respectively), and if relapsed, encouragement to set a new quit date (34/44, 77% and 27/31, 87%, respectively). Few apps provide support regarding relapse in the form of a reminder that quitting takes practice (7/44, 16% and 1/31, 3%, respectively). Informational apps hardly arranged follow-ups.

### Quality (MARS Score) of the Apps by Type

Table 3 shows the mean MARS scores by app type. The mean MARS scores of the combined, multifunctional, and informational apps were comparable at 3.64, 3.26 and 3.0, respectively. The mean scores on the 4 MARS dimensions were calculated. The functionality dimension scores were the highest among the subscores (3.97, 3.83, and 3.86 for combined, multifunctional, and informational type, respectively) and scores on the engagement dimension were the lowest (3.52, 3.1, and 2.23 for combined, multifunctional, and informational type, respectively). Information quality score was the lowest in the multifunctional type (3.04). For the combined and multifunctional type, the mean scores on all dimensions were 3 or higher, whereas they were less than 3 for the informational type, except in functionality. The difference in all the dimension scores by type was statistically significant except the functionality score.

**Table 3:** Quality of the apps by type (N=104).

Mobile App Rating Scale component scores			Combined type (n=44), mean (SD)		Multifunctional type (n=31), mean (SD)		Informational type (n=29), mean (SD)		*F* test *(df)*		*P* value
**Total score**			3.64 (0.38)		3.26 (0.48)		3.0 (0.32)		29.21 (2)		<.001
	Engagement	3.52 (0.57)		3.1 (0.57)		2.23 (0.39)		68.65 (2)		<.001	
	Functionality	3.97 (0.53)		3.83 (0.58)		3.86 (0.42)		1.04 (2)		.36	
	Aesthetics	3.60 (0.69)		3.08 (0.78)		2.97 (0.52)		10.37 (2)		<.001	
	Information quality	3.53 (0.56)		3.04 (0.47)		2.93 (0.38)		15.52 (2)		<.001	

### App Features Affecting Quality (MARS Score) or Content and Function (Content Score)

Table 4 shows the results of a multivariate analysis of app features, MARS scores, and content scores. Feedback channel with developer and developer sector had a significant impact on both scores. Both MARS and content scores were higher when feedback with a developer was possible within the app compared to that when feedback was only available at the app market level. The MARS score was higher for apps developed as a collaboration between government and commercial institutions compared to that when the developer was unknown. The content score was higher for apps developed by government or a university or commercial institution compared to that when the developer was unknown. Platform type was found to have a significant impact on MARS score, with iPhone apps having higher MARS scores than Android apps.

**Table 4:** Multiple regression analysis of quality (Mobile App Rating Scale score) or content and function (content score) (N=104).

Category, items		Mobile App Rating Scale score				Content score		
		β	SE	*P* value	β		SE	*P* value
Intercept		2.91	0.07	<.001	9.22		0.95	<.001
**Developer sector**								
	Government	.17	0.21	.42	6.98		2.67	.01
	University	.28	0.20	.17	7.14		2.60	.01
	Government with commercial	.52	0.23	.03	4.87		2.99	.11
	Nongovernmental organization	.31	0.28	.27	2.69		3.61	.46
	Commercial	.12	0.09	.18	3.50		1.17	<.001
	Unknown	Ref^a^	Ref	Ref	Ref		Ref	Ref
Affiliation of developer with health care professionals		.11	0.13	.38	2.82		1.66	.09
Affiliation of developer with non–health care professionals		Ref	Ref	Ref	Ref		Ref	Ref
**Platform**								
	Both	.17	0.11	.13	1.40		1.41	.33
	iPhone	.26	0.11	.02	–.20		1.40	.88
	Android	Ref	Ref	Ref	Ref		Ref	Ref
**Payment type**								
	Prepaid	–.12	0.13	.36	–.26		1.62	.87
	In-app purchase	.21	0.10	.04	1.40		1.34	.30
	Free	Ref	Ref	Ref	Ref		Ref	Ref
**Feedback channel with developer**								
	Within the app	.35	0.10	<.001	3.96		1.24	<.001
	Contact information of developer provided	.17	0.13	.19	2.01		1.67	.23
	Market level only	Ref	Ref	Ref	Ref		Ref	Ref

^a^Ref: reference value.

## Discussion

### Main Results

This study classified existing smoking cessation apps into 3 types and then evaluated their content (or functions) and quality. The characteristics associated with content and MARS scores were also analyzed. Combined type apps had the highest content and MARS scores among the 3 app types. Multifunctional type apps had higher MARS scores than informational type apps. Content and function analysis showed that multifunctional apps better represented the function-related components of “ask, assist, and arrange follow-ups” than did informational apps. On the contrary, informational apps better represented the information-related components of “advise and assess” than did multifunctional apps. Some previous studies that analyzed the content of smoking cessation apps by using the 5As guideline reported that very few apps actually conformed to the guideline [12,17,18]. In one such study, the percentage of apps consistent with the (modified) 5As guideline did not exceed 50% except for the “assist” dimension [20]. In a study analyzing smoking cessation apps using MARS, a mean MARS score of 2.88 was reported [24]. The apps analyzed in our study better adhered to the 5As guideline and had higher MARS scores than those reported in previous studies [12,17,18,20,23], possibly because the functions of the apps may have been improved in the interim. Other studies used clinical guidelines to analyze app content [12,17,18]; therefore, guideline adherence may have been lower than that when using guidelines specifically designed for apps [20]. It has been shown that decision-aid apps with multiple functions such as motivational messages, a quit diary, and a quitting benefits tracker but including scant information on quitting strategies are more effective for smoking cessation than information-only apps [14]. Another study found that evidence-based apps with customized functions and information were more effective than a self-help booklet [15]. Our results support these studies where the combined apps, which are similar to decision-aid apps and evidence-based apps, had higher scores than informational apps.

Mobile-based interventions have advantages over standard interventions for smoking cessation [25]. One such advantage of smartphone apps is the potential to deliver a user-centered interactive intervention. Combined apps are considered able to better exploit this advantage, and the content scores and MARS scores of this type of apps support that view. Multifunctional apps are somewhat in line with this. In this study, the combined type had the best general characteristics followed by the multifunctional and informational type. Combined apps were more likely to be developed by trusted institutions (government or universities) or health professionals and to not include advertisements and be available for free, followed by multifunctional and informational apps.

Regression analysis showed that the sector in which a developer is situated and the feedback channel with developers are important. Our findings suggest that governments or universities should ideally be the creators of smoking cessation apps. Most of the apps produced by governments and universities were of the combined type, which had high content and MARS scores. In a previous study [24], 3 of 6 apps that received high scores were developed by the government; the other 3 were produced by research institutions. In detail, the regression analysis showed that government- or university- or commercial institution–developed app content scores were high. The MARS scores were high for apps developed collaboratively by government and commercial institutions. Although not described in the results, 4 apps created via such collaborations had a mean MARS score of 3.96, which is above the average score of combined type apps (3.64). Apps developed collaboratively by government and commercial institutions benefit from the reliable information provided by the former and the technical expertise of the latter.

In this study, apps providing an option for feedback with a developer had higher content and MARS scores. Further, users themselves provide more feedback when they are able to do so directly within the app, where such feedback can help improve app content and MARS scores. Although the content and MARS scores do not necessarily indicate smoking cessation efficacy, the interaction between users and developers is beneficial when creating a smoking cessation app or other mHealth apps; therefore, a feedback function should be included.

### Secondary Features Offered to Users

Informational apps had generally low content scores because they provide only information and have no functions. The low MARS scores of informational apps may have been influenced by the high proportion of unknown developers for these apps (24/29, 83%); informational apps can be created easily by almost anyone. It is easy to obtain general information pertaining to smoking cessation from the internet, and nameless developers have no responsibility to maintain or improve their apps. Although there were more than 49 apps of informational type in the finally selected apps, more than 20 apps were excluded from the final analysis. App instability [24] can be an issue—also due to an absence of management by unknown developers. The biggest problem with respect to informational apps was that they were mostly copies of other apps. Ten informational type apps included in the final analysis were copied apps that had different developers.

The large market share of counter and hypnosis apps is also problematic. Counter apps estimate the money saved by smoking cessation or the number of cigarettes not smoked. Hypnosis apps promote smoking cessation through video- or audio-based hypnosis. Of the 394 apps downloaded after the screening phase, 157 were counter apps (40%). Since hypnosis apps were excluded at the screening phase, the exact number of such apps was not counted, but they also appeared to have a large market share. In fact, when searching for apps using the term “quit smoking,” 11 apps (6 counter and 5 hypnosis type) in Google Play and 6 apps (5 counter and 1 hypnosis type) in the App Store were noted among the top 20 apps in each market. Single-function apps might be useful for users whose digital literacy might be limited or who are prescribed the apps by a health care provider for a specific purpose. However, their function is quite insufficient for the general public. A 2011 study reported low content scores for single-function apps (counter and hypnosis apps) [17]. The result of a Cochrane review showed that the effectiveness of hypnosis for smoking cessation is not clear [26]. Further, it has been found that the transference relationship between the therapist and patient influences the smoking cessation efficacy of hypnosis, but it is difficult to build such a relationship through an app [26]. In a recent study, the effect of a meditation app for quitting smoking was not revealed [27]. If problematic apps (made by an unknown developer or copied) and single-function apps have a large market share, users may find it difficult to access better apps. In this study, we were able to identify the problematic apps as above through the process of the app exclusion.

### Limitations

First, owing to app volatility, many apps were suspended during the analysis. High-quality apps should be maintained through collaboration between government and commercial institutions. Second, as the purpose of this study was to compare apps by function, single-function apps, which have only 1 function (eg, counter or hypnosis), were excluded. Since those are not applicable in many items of evaluation tools, evaluation has no meaning. In future research, it will be possible to analyze and evaluate only these apps. Lastly, although we identified apps with high MARS scores and with a large amount of content as recommended by the Clinical Practice Guideline for Treating Tobacco Use and Dependence, we cannot definitively conclude that these apps would be effective with respect to smoking cessation. Further studies should be conducted to confirm the smoking cessation efficacy of different classes of apps.

### Conclusions

This study can be a guide for users and developers of smoking cessation apps. This is the first study to evaluate the content and quality of smoking cessation apps by classification. The combined type had higher-quality content and functionality than the multifunctional type and the informational type. However, the other two types also had their own advantages. The classification of the apps can help users choose the appropriate type of smoking cessation app for their needs. The identification of characteristics that affected the app scores in this study may help in the development of a smoking cessation app (development collaboration between health professionals, academic researchers and industry, inclusion of a communication option with a developer within the app, etc). Additionally, we identified that problematic apps such as those made by unknown developers or copied and single-function apps had a large market share. This could promote discussion on the possible regulation of problematic apps. Public health–related apps should be more rigorously examined before being released to the market. In summary, our results will contribute to the use and development of better smoking cessation apps.

## Appendix

Multimedia Appendix 1 App list.

## Abbreviations

5As: ask, advise, assess, assist, and arrange follow-ups
MARS: Mobile App Rating Scale
mHealth: mobile health
RR: relative risk
