# Supplementary material for: Classification of Smoking Cessation Apps: Quality Review and Content Analysis
Source: JMIR Mhealth Uhealth. 2022 Feb 17;10(2):e17268. doi: 10.2196/17268 (PMC8895289; doi:10.2196/17268)
Supplement: Multimedia Appendix 1 [file mhealth_v10i2e17268_app1.pdf]

Appendix 1. App list

Type	Num ber	Name	Developer	Content score	MARS score	Plat form	Cost (won)	Affiliation of developer¶	Feed back§
**Combind type**	1	quitSTART -Quit Smoking	ICF International	28	3.59	Both	0	1	2
	2	No Butts	University of California, San Diego	28	4.26	iPhone	0	1	1
	3	Alex AI - Quit smoking	Alex Therapeutics	28	4.54	Both	1500~ 80000*	5	1
	4	Quit smoking , dept of health	tobacco control office, department of health	27	3.44	Both	0	1	2
	5	Quit Right	Stop Smoking Strategies, LLC	26	3.65	Both	0	5	2
	6	Stop-tabac	Université de Genève	26	3.71	Both	0	2	2
	7	QuitNet	MeYou Helth	26	3.99	iPhone	0	5	1
	8	Beat Smoking - Quit Smoking	Prodocity	25	3.72	Both	1100*	5	1
	9	SmokeFree Baby - Stop Smoking	British Apps	25	4.22	Both	0	2	1
	10	My Quitbuddy	Quit Now: My QuitBuddy	25	4.56	Both	0	3	1
	11	Quit Genius - Best way to quit smoking for good	Digital Therapeutics	25	4.26	Both	12000~ 18000*	5	1
	12	Sacabo	Manuel Conde	25	3.97	iPhone	0	1	2
	13	QuitNow	Optum Inc.	25	3.49	Android	0	5	1
	14	Stay Quit Coach	US Deparment of Veterans Affairs	24	3.55	Both	0	1	2
	15	SmokeFree - quit smoking slowly	MotiveBite Studio	24	3.52	Android	0	5	1
	16	Stop tobacco mobile trainer, quit smoking app free	Iteration mobile & vialsoft apps	23	3.85	Both	7500	2	2
	17	QuitGuide - quit smoking	ICF International	23	3.41	Both	0	1	2
	18	Craving To Quit!	Claritas MindSciences LLC	23	3.92	Both	28000*	3	1
	19	SiS - Smiling Instead of Smoking	CBITs	23	3.46	Android	0	2	3
	20	Quit Smoking in 101 days	Ultimate Party Apps	21	4.17	Both	5500*	5	1
	21	Quitify	SpanishApps	21	3.37	Android	0	6	3
	22	Quit4goodlife (Smoking Cessation - Quit Smoking)	technology Digest inc	20	3.54	Both	1100*	5	3
	23	GFH lite	Dandachli, LLC	20	3.89	iPhone	0	5	1
	24	Smoke Free, stop smoking now and quit for good	The Quit Smoking Specialists	19	3.35	Both	1300~ 6905*	5	1

	25	Pare - Quit Smoking Cigarettes	OneSci Inc	19	3.89	Both	0	5	1
	26	Flamy – quit smoking & become a non-smker	Offlinefirst	19	3.74	Android	1000~ 9500*	5	1
	27	Breathe easy smoking cessation	Push Interactions, Inc.	18	3.44	Both	0	3	3
	28	Smoke Free 28 (SF28) Quit Smoke...	British Apps	18	3.40	Both	0	2	2
	29	My Quit Luton	British Apps	18	2.97	Both	0	2	1
	30	SmokeLess Quit	Julio Velasquez	18	3.66	iPhone	0	5	1
	31	Quit smoking	Andeko	17	3.39	Android	1839*	6	3
	32	3-2-1 Quit Smoking	Snappademics LLC	17	3.65	iPhone	0	5	1
	33	Quit for you - Quit for two	Carbon Media	17	3.96	iPhone	0	5	3
	34	NTSmoking: Quit smoking right now	Gravity Ecom	17	3.35	Android	0	5	3
	35	Smoke – quit	NikNormSoft	16	2.90	Android	0	6	3
	36	Break it Off	Canandian Cancer Society	16	3.47	iPhone	0	1	2
	37	Quit tobacco	Memsta apps	15	3.31	Both	999*	5	3
	38	Puff Away - Stop Smoking Today	Hexpress Healthcare Ltd	15	3.70	iPhone	0	5	3
	39	Quit for us	Diva Creative	15	3.94	iPhone	0	3	3
	40	IQ Today - Smoking	Jarrod Kanizy	15	3.15	iPhone	3289*	6	3
	41	Arretez de fumer - stop tobacco	Best App Made With Love	14	3.32	Both	5159*	6	3
	42	Stop Smoking	Fort Sanders Foundation	14	2.78	Android	0	5	2
	43	No Smoking Calender	App Diggity, LLC	13	3.45	iPhone	1199*	5	3
	44	Smoke Free: Stop, Quit, No Smoking – Quit Tracker	Wellness Labs	13	3.36	Android	6000*	5	1
**Multi-functional type**	45	Butt Out Quit Smoking Forever	ellisapps Inc.	23	2.56	Both	3900	5	1
	46	Let's quit smoking	4wl.Apps	19	3.25	Android	0	6	1
	47	Stop Smoking - EasyQuit free	Mario Hanna	19	3.97	Both	0	6	1
	48	LIVESTRONG MyQuit Coach	LIVESTRONG.COM	18	3.93	iPhone	1199*	4	1
	49	Stop Smoking - quit Smoking, be smoke free	The Quit Smoking Professtionals	18	3.90	Android	999~ 1700*	5	1
	50	Qwidder : Quit Smoking	Qwidder Inc.	17	4.13	Both	2700*	5	1
	51	Qwit (Quit Smoking)	Team Geny	16	3.60	Android	1000*	5	2
	52	Quit Smoking Buddy	HQmedia	16	3.79	Both	0	5	1
	53	Stop Smoking[1]	AB Mobile Apps	16	2.79	Both	0	5	1

	54	Quit Smoking[1]	AB Mobile Apps	16	2.79	Both	0	5	1
	55	Quitter	Guardanis	16	2.71	Android	0	5	3
	56	I Don't Smoke - free way to quit smoke	Theros	16	3.59	iPhone	1199*	6	1
	57	Quit Smoking<br>- No smoking day	PIONE STDIO	15	3.68	Android	0	5	3
	58	QuitNow!	Fewlaps	14	3.78	Android	4335*	5	1
	59	Get Rich or Die Smoking	Tobias Gruber	14	3.06	Android	0	6	1
	60	Quit Smoking<br>- Stop for Good Health & Track[2]	Beta Garden	14	2.66	Android	0	5	3
	61	Quit Smoke[2]	Taimoor Suleman	14	2.66	Android	0	5	3
	62	Nichtraucher[2]	Foss Android Development	14	2.66	Android	0	5	3
	63	Quit smoking[2]	Vladislav Zhirnov	14	2.66	Android	1100*	5	3
	64	Smoke FREE<br>- Non Smoking	sg-pages	14	2.89	iPhone	2409*	6	3
	65	Quit Smoking: Cessation Nation	Ron Horner	13	3.50	Android	0	4	2
	66	ExSmoker	Antonio Sanchez Diaz	13	3.23	Android	0	5	3
	67	Natural Non -Smoker	Supriya	12	3.43	iPhone	0	6	2
	68	Quit-Smoking Coach Free	Brainlag Studios	12	2.88	Android	0	6	3
	69	DipQuit: Quit Dipping Smokeless Tobacco	Mirsad Hasic	12	3.84	Both	5807	6	3
	70	Quit My Way	SMART ADDICTION TECHNOLOGIES INC.	11	3.16	Both	4700	5	3
	71	Quit smoking for free	ZeroZig	11	3.16	Android	0	5	3
	72	SmokeTab	Avenye Technologies Inc.	11	2.83	iPhone	1199	5	3
	73	Smoking Cessation	MAGNA HEALTH SOLUTIONS	9	2.96	Both	0	6	3
	74	Quit My Way	SMART ADDICTION TECHNOLOGIES INC.	8	3.36	Both	4700	5	3
	75	Smoking Cessation Program<br>- binaryGURU Apps	Sudeep Chandra, Binary Consulting & Sof…	6	3.66	Both	3500*	5	1
**Informatio-nal type**	76	Stop Smoking 3D[3]	World Cloud Ventures Sdn Bhd	11	3.03	iPhone	0	6	3
	77	How to Quit Smoking	Boorhan	10	2.62	Android	0	6	3
	78	Stop Smoking in 5 Days Free[3]	Analia Pedulla	10	3.03	iPhone	12000	6	3
	79	Stop Smoking in Five Days - Leopar…[3]	daniel gonzalez	9	3.03	iPhone	12000	6	3

	80	Stop Smoking in Five Days - Five days…[3]	daniel gonzalez	9	3.03	iPhone	12000	6	3
	81	You Can Quit Smoking	Insplisty	9	3.12	Android	0	5	2
	82	My Last Cigarette Chanllenge - Stop…[3]	The Jones Kilmartin Group	9	3.03	iPhone	3900	5	3
	83	Free Life: Stop Smoking -Quit To…[3]	daniel gonzalez	9	3.03	iPhone	5900	6	3
	84	Stop Smoking X[3]	Stop smoking and stay quit	9	3.03	iPhone	12000	6	3
	85	Better Body : Quit Smoking	mmotio	9	3.83	iPhone	6039	5	1
	86	Stop Smoking 2016[3]	daniel gonzalez	9	3.03	iPhone	12000	6	3
	87	Quit smoking	AccessGames	8	3.78	Android	0	6	3
	88	Quit Now Studio- Stop Smoking Tod…[3]	Stop Smoking App	8	3.03	iPhone	3900	6	3
	89	Smoke Free - quit smoking plus	AXON	8	3.13	Android	0	6	2
	90	Quit Smoking Now: Smoking Cessation[3]	Kenneth Edwards	8	3.03	iPhone	3900	6	3
	91	Stop Smoking (How To Guide)	Quotes & Facts	7	2.90	Android	0	6	3
	92	Quit Smoking Secrets[4]	PicLabs	7	3.00	Both	0	6	3
	93	Quit Smoking Secrets[4]	MobyiApps	7	3.03	Both	0	5	3
	94	The Best way to quit smoking	BigBoss Studio	7	2.71	Android	0	6	3
	95	Te Ohu Auahi Mutunga - TOAM	Konnect Applications	6	3.26	iPhone	0	1	1
	96	How To Quit Smoking	Leon Technologies	6	3.32	Android	0	6	3
	97	Quit Smoking Guide	Rowan Saturnin	6	2.99	Android	0	6	3
	98	Quit Smoking Tips	KrishMiniApps	6	2.67	Android	0	6	3
	99	Quit Smoking	Nature Healthy Care	5	2.85	Android	0	6	3
	100	Quit Smoking	Free Mobile Shop Apps	5	3.07	Android	0	6	3
	101	how to quit smoking	Best free Apps 2018	5	2.94	Android	0	6	3
	102	Quit Smoking	Expert Health Studio	4	2.69	Android	0	6	3
	103	Quit Smoking	VorteX	4	2.13	Android	0	6	3
	104	Quit Smoking	Canada Dev Apps	3	2.70	Android	0	6	2

[1],[2],[3],[4]Copied app

* price in-app purchase

¶ 1: Government 2: University 3: Government+Commercial 4: NGO 5: Commercial 6: Unknown

§ 1: Possible inside the app 2: Providing contact information inside the app 3: Only possible in app market
